# Attention-enhanced residual autoencoder for NIR spectral feature extraction and classification of grain varieties

**DOI:** 10.1038/s41598-025-17676-w

**Published:** 2025-09-24

**Authors:** Abel Chernet Kabiso, Cheng-Hao Ko

**Affiliations:** Graduate Institute of Automation and Control, National Taiwan University of Science and Technology (NTUST), No. 43, Section 4, Keelung Rd, Da’an District, Taipei, 10607 Taiwan

**Keywords:** Portable NIR spectroscopy, Grain classification, Autoencoder, Attention mechanisms, PCA, Plant sciences, Engineering, Mathematics and computing

## Abstract

Accurate identification of grain cultivars is critical for improving crop yields, streamlining agricultural workflows, and ensuring global food security. Near-infrared (NIR) spectroscopy offers a rapid, non-destructive solution for grain classification. However, its effectiveness hinges on extracting meaningful spectral features. We propose SpecFuseNet, an attention-enhanced residual autoencoder, as a lightweight deep learning model for extracting NIR spectral features and classifying grain varieties. The encoder integrates Fused Efficient Channel Attention (FusedECA) and a Spectral Residual Gate (SRG) to extract informative spectral features, while a mirrored decoder enables robust spectral reconstruction. This architecture supports both spectral reconstruction and cultivar classification, with robust performance and minimal complexity. We evaluated SpecFuseNet on three NIR datasets: barley (1,200 samples, 24 varieties), chickpea (950 samples, 19 varieties), and sorghum (500 samples, 10 varieties) using stratified 5-fold cross-validation. The model achieved classification accuracies of 89.72%, 96.14%, and 90.67%, respectively, outperforming PCA-based machine learning models (SVM, Random Forest, XGBoost) and deep learning baselines such as standard Autoencoder (AE) and Convolutional Sparse Autoencoder (CSAE). These results demonstrate SpecFuseNet’s potential as a fast, interpretable, and deployable solution for real-time classification in field-based and resource-limited settings, with a lightweight design that enables deployment on portable or smartphone-connected NIR spectrometers, supporting sustainable and precise agricultural practices.

**Supplementary Information:**

The online version contains supplementary material available at 10.1038/s41598-025-17676-w.

## Introduction

The accurate classification and monitoring of grain quality play a pivotal role in ensuring food security and supporting modern agricultural practices. Conventional methods such as visual inspection, DNA analysis, and field-based evaluations remain widely used but are often labor-intensive, subjective, and time-consuming^1^. However, limitations including sample degradation, environmental influences, and prolonged turnaround times underscore the need for efficient, rapid, and reliable alternatives. Furthermore, the visual similarity among grain varieties further complicates identification, often necessitating the use of sophisticated imaging systems.

To overcome these limitations, spectroscopic techniques, particularly near-infrared (NIR) spectroscopy, have gained traction as viable alternatives. NIR spectroscopy offers a rapid, non-destructive means of detecting both the physical and chemical properties of agricultural samples. Its applications span multiple domains, including food safety, pharmaceuticals, and environmental monitoring^2–7^. With ongoing advances in miniaturization and cost-effectiveness^8–10^, portable spectrometers now enable real-time, in-field analysis^11,12^. Nevertheless, variations in grain morphology, such as differences in size, shape, and orientation, continue to influence spectral signatures and introduce challenges for classification accuracy^13^. Meanwhile, rapid progress in computing and data availability has propelled deep learning’s expansion into areas such as image analysis, speech processing, and natural language processing.

Recent advances in machine learning and deep learning have significantly accelerated the adoption of NIR spectroscopy in agriculture by enhancing feature extraction and classification. Classical techniques like Principal Component Analysis (PCA), Linear Discriminant Analysis (LDA), Wavelet Transform (WT), Independent Component Analysis (ICA), and Partial Least Squares (PLS) have been used to extract informative features from spectral data^14^. However, these linear or shallow approaches often fail to capture the nonlinear and high-dimensional relationships inherent in spectral signals. In contrast, particularly convolutional neural networks (CNNs) based deep learning methods offer superior performance by enabling hierarchical, automatic feature extraction^15^. CNNs have been successfully applied to various agricultural tasks including crop disease detection, yield estimation, and grain classification^16–20^.

While basic CNNs improve performance, more complex architectures have been introduced to further enhance spectral classification. Inception modules^21,22^, residual networks^23–26^, and attention mechanisms^27–29^ enable multi-scale feature extraction, emphasize relevant spectral channels, and improve model interpretability. More recently, autoencoders have been explored for their ability to learn compact and informative spectral representations. Fully connected and convolutional autoencoders^30–33^ have demonstrated promising results, especially when combined with residual learning and attention mechanisms to boost robustness and convergence. A machine learning-based framework integrating uncertainty quantification for crop recommendation is proposed to make the decision-making process more robust and sustainable^34,35^. Transformer models have also been explored for NIR spectroscopy, with studies evaluating the impact of preprocessing techniques and machine learning algorithms on variety classification^36^.

Despite these advances, existing models still struggle to fuse shallow and deep spectral features effectively or to dynamically emphasize the most informative channels without incurring high computational cost. While residual connections alleviate vanishing gradients, simple skip paths can propagate noise and dilute key spectral features^37^. To address these issues, recent work has explored channel-attention mechanisms (e.g., Squeeze‐and‐Excitation, Efficient Channel Attention) and gated residual strategies to boost feature selectivity and efficiency^38–42^. However, while these approaches improve performance, they do not fully address challenges related to noise propagation and the effective integration of local and global spectral features. Recent attention-based deep learning models^43–46^ have demonstrated that combining convolutional layers for local encoding with transformer-style attention modules significantly improves spectral modeling.

Building upon these insights, we present SpecFuseNet, an architecture that integrates a Convolutional Autoencoder (CAE) with fused Efficient Channel Attention (FusedECA) and spectral residual gate (SRG) modules. Our attention-residual framework advances chemometric modeling by dynamically emphasizing discriminative spectral features, improving both feature extraction and classification performance. The FusedECA module combines global average pooling (GAP) and global max pooling (GMP) using lightweight 1D CNNs to extract complementary spectral features without introducing significant complexity. The SRG dynamically modulates residual connections, refining spectral representations and suppressing irrelevant patterns. The encoder consists of three convolutional blocks, each augmented with FusedECA and SRG, while the decoder mirrors this structure using deconvolution layers for spectral reconstruction. The feature embeddings extracted by the encoder are processed by a classifier consisting of a flattening layer followed by two fully connected dense layers for grain variety classification. To validate the effectiveness of SpecFuseNet, we conduct extensive experiments on a public NIR spectral dataset covering three grain types: barley (1,200 spectra from 24 varieties), chickpea (950 spectra from 19 varieties), and sorghum (500 spectra from 10 varieties). The model is benchmarked against classical machine learning methods using PCA-based features (SVM, Random Forest (RF), XGBoost), as well as standard Autoencoder and Convolutional Sparse Autoencoder (CSAE) frameworks. This comparative study establishes SpecFuseNet as a robust framework for accurate and efficient grain variety feature extraction and classification, offering a promising solution for agricultural and food industry applications where precise spectral data analysis is critical. The objectives of this research work are as follows:

i. Develop and implement SpecFuseNet, a deep learning model for efficient spectral feature extraction and classification of NIR spectra from grain varieties, employing a hybrid learning strategy with stratified 5-fold cross-validation.
ii. Design and integrate the FusedECA and SRG modules within the CAE framework to enhance feature extraction and dynamically modulate residual connections.
iii. Evaluate the performance of SpecFuseNet against classical PCA-based features combined with benchmark classifiers (SVM, RF, XGBoost) and baseline models (AE and CSAE) using multiple metrics including accuracy, precision, recall, and F1 score.

## Materials and methods

### Dataset

The dataset used in this study was collected using the SCIO Consumer Edition, a miniaturized NIR spectrometer developed by Consumer Physics. This portable apparatus functions via a smartphone application and necessitates an internet connection to facilitate the transmission of the obtained spectral data to a remote server. It encompasses a wavelength spectrum from 740 to 1070 nanometers, yielding 331 spectral variables. All grain samples were consistently scanned under similar conditions to ensure measurement reliability. The dataset comprises NIR spectra collected from grain samples of 50 varieties—24 barley, 19 chickpea, and 10 sorghum cultivars—prepared and collected by the Ethiopian Institute of Agricultural Research (EIAR) in June 2017^16^. For each variety, 50 grain samples were obtained, resulting in a total of 1,200 barley grain samples, 950 chickpea grain samples, and 500 sorghum grain samples. To eliminate the potential influence of seed age, all grains were harvested within the same year.

**Fig. 1:**
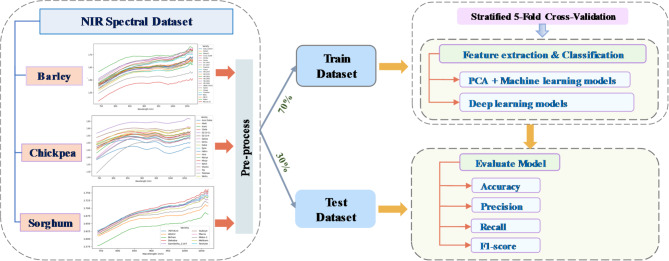
Data analysis workflow for grain cultivar classification.

In earlier studies on this dataset^16,36^, machine learning and deep learning approaches were applied using various preprocessing methods on the raw spectra. In this study, we preprocessed the raw spectra using the SG filter with a fixed window size 5 points, a second-degree polynomial, and the first derivative. This method effectively enhanced signal quality by smoothing noise while preserving key spectral features. Following this, standardization was applied to scale the data to zero mean and unit variance, further improving the consistency of the input features. To ensure a robust and unbiased evaluation of the model’s predictive performance, stratified 5-fold cross-validation was employed. This approach partitions the dataset into five folds while maintaining the original class distribution in each fold. In each iteration, four folds were used for training and one for validation, ensuring balanced class representation, reducing performance variance, and providing reliable, generalizable results. The detailed data analysis workflow is illustrated in Fig. 1.

### SpecFuseNet architecture details

**Fig. 2:**
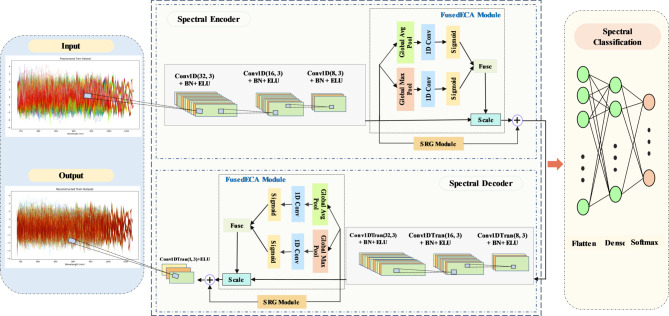
SpecFuseNet spectral feature extraction and classification network architecture.

Conventional spectral feature extraction methods, such as PCA, ICA, linear LDA, and WT, facilitate the projection of the original dataset into an alternative feature space while concurrently retaining critical information. In contrast, deep learning-based approaches for spectral feature extraction include supervised models, such as CNNs and recurrent neural networks (RNNs), as well as unsupervised methods like AEs^33^. The feature reduction techniques of AE network composed of two symmetric parts: an encoder and a decoder. The encoder maps high-dimensional input data into a lower-dimensional latent space, while the decoder reconstructs the original input from this compressed representation. While shallow networks are limited in their ability to extract complex features, deeper architectures can significantly reduce the complexity of function representation and lower the amount of training data required. In this study, the SpecFuseNet model is proposed, integrating a CAE with FusedECA and SRG modules. The encoder part is composed of three convolutional layers, each enhanced with the FusedECA and the SRG modules, while the decoder symmetrically mirrors this structure with deconvolutional layers, and ends with an output deconvolution layer. The proposed architecture was developed to facilitate effective spectral feature extraction and accurate classification of grain varieties, as illustrated in Fig. 2.

The proposed SpecFuseNet model integrates four main components to achieve effective spectral feature extraction, reconstruction, and classification in an end-to-end learning framework. The input-output module first preprocesses the raw spectral data to enhance quality and ensure training stability through normalization and noise reduction. The spectral encoder network then extracts hierarchical features using a series of convolutional layers, augmented with batch normalization (BN) for stable training and Exponential Linear Unit (ELU) activations for improved nonlinear representation. The FusedECA module is incorporated to adaptively recalibrate channel-wise features while maintaining computational efficiency, and the SRG modules dynamically modulate skip connections to preserve critical information during deep feature extraction. The spectral decoder network mirrors the encoder’s structure, employing transposed convolutions to reconstruct the input spectra with high fidelity by progressively reversing the encoding process, and is followed by an output deconvolution layer. Beyond reconstruction, the decoder serves as an auxiliary supervision branch that reinforces the learning of meaningful spectral representations. By guiding the encoder to preserve detailed spectral characteristics, the reconstruction task regularizes the feature space and prevents overfitting to purely discriminative features. This added constraint improves the generalization of the encoder and enhances the discriminative quality of the learned features, ultimately benefiting classification performance. Finally, the classification network processes the encoded features through a flattening layer followed by two fully connected layers, with a Softmax-activated output layer generating class probabilities for multiclass discrimination. By unifying feature learning, reconstruction, and classification within a single optimized framework, the SpecFuseNet model ensures robust performance in handling complex spectral datasets while minimizing information loss and maximizing discriminative power.

**One dimensional convolutional layer (Conv1d)**: In CNNs, convolution layers play a central role in extracting meaningful features^47^. These layers apply operations that are both linear and translation-invariant, helping to highlight key patterns while reducing irrelevant noise. This process ultimately strengthens important signals and improves model performance. For a spectral input sequence: $\:X={x}_{1},{x}_{2},.\:.\:.\:.\:.\:{x}_{n}$, the one-dimensional convolution operation defined as:

1 $$\:y\left[i\right]=\:\sum\:_{j=0}^{k-1}w\left[j\right]\cdot\:x\left[i\times\:s+j\right]+b$$

In this equation, $\:y\left[i\right]$ denotes output sequence of $\:i$th element, k represents the kernel size (set to 3), $\:w\left[j\right]$ represents the $\:j$th weight of convolution kernel, $\:s$ denotes the stride (set to 1), and $\:b$ is for bias. With same padding, the output sequence has the same length as the input sequence, which is expressed as: $\:Y={y}_{1},{y}_{2},.\:.\:.\:.\:.\:{y}_{n}$. After each convolution layer, BN is applied to speed up the training and enhance feature extraction. Additionally, a non-linear activation function is employed, which enhances the model’s capacity to learn complex patterns in the spectral data.

#### FusedECA module

**Fig. 3:**
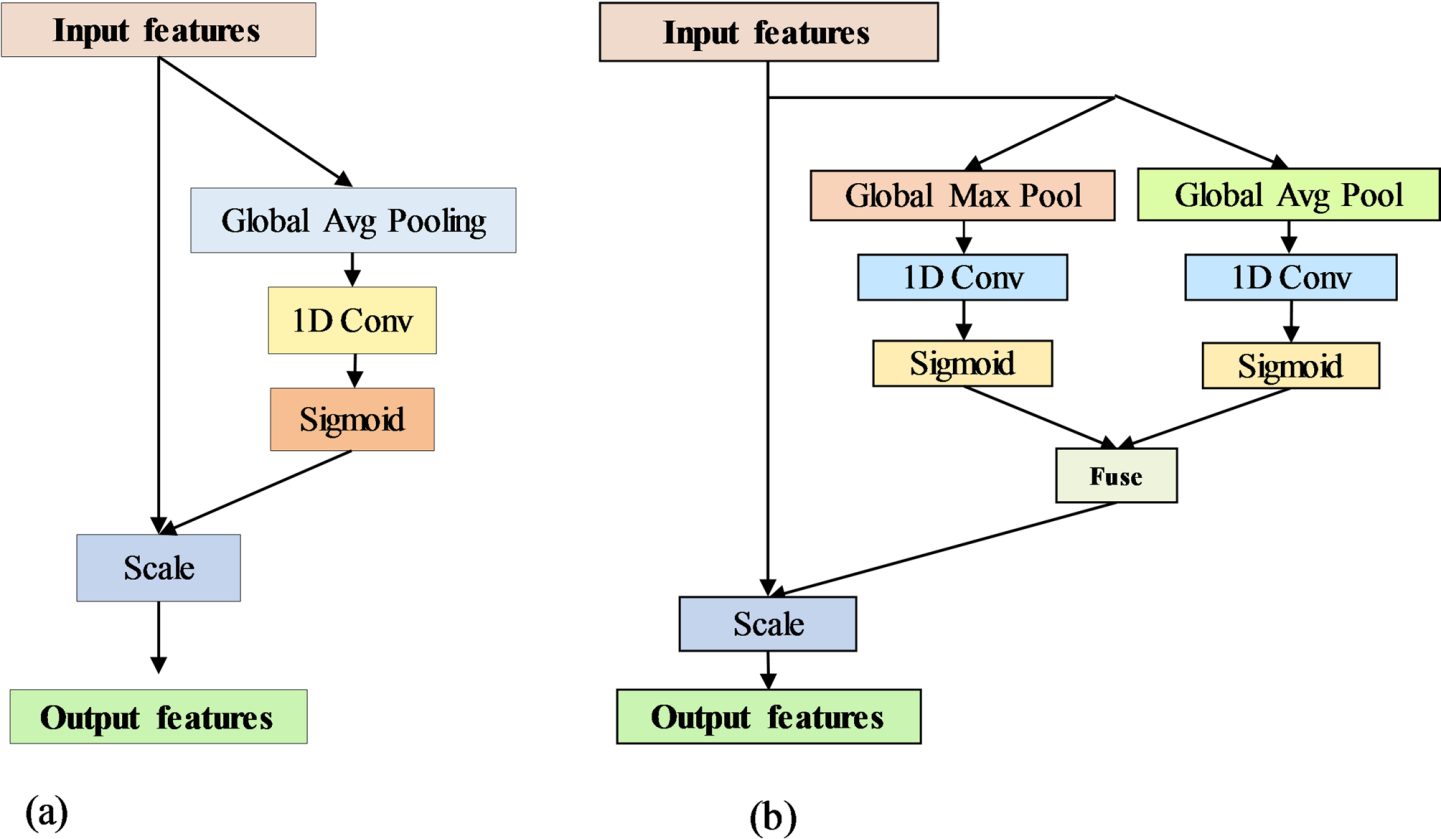
(**a**) Original ECA module using GAP-based attention. (**b**) Proposed FusedECA module integrating both GAP and GMP for enhanced spectral feature selection.

To improve critical spectral feature extraction, the FusedECA module is incorporated within the CAE network. In spectral data, relevant information is often sparsely distributed across wavelengths, and subtle local variations can be crucial for accurate classification. However, the original Efficient Channel Attention (ECA) module^41^, which primarily relies on GAP to model channel dependencies, may not fully capture the diverse and localized nature of spectral signals, as shown in Fig. 3a. To address this limitation, the proposed FusedECA module integrates both GAP and GMP operations in parallel, producing two complementary 1D descriptors per channel—one capturing average activation trends and the other representing the strongest localized signal responses.

Each descriptor is then passed through a lightweight 1D convolutional layer followed by a sigmoid activation to produce two channel-wise attention maps. These attention maps are fused using an element-wise maximum operation, generating a unified attention vector that preserves the most informative responses from either pooling branch. This fused attention vector is used to recalibrate the original input feature map through element-wise multiplication, allowing the network to selectively emphasize channels that contain either consistently strong or uniquely prominent spectral patterns. By leveraging both pooling strategies and applying efficient convolutional operations, the FusedECA module provides a robust yet lightweight solution for enhancing discriminative spectral features while suppressing irrelevant information. The complete mechanism is illustrated in Fig. 3b.

The FusedECA module’s operations are mathematically defined as follows. Given an input feature map $\:x\varepsilon {\mathbb{R}^{C \times \:m}}$, the module first compresses the spectral dimension separately using GAP and GMP, generating two distinct 1D descriptors that capture the mean and the most prominent signal patterns, respectively:

2 $$\:{z}_{k}^{GAP}=\frac{1}{m}\sum\:_{i=1}^{m}{x}_{k}\left(i\right),\:k=1,\dots\:..,C$$

3 $$\:{z}_{k}^{GMP}={max}_{i=1}^{m}{x}_{k}\left(i\right),\:k=1,\dots\:..,C$$

Next, to model channel dependencies within each descriptor, a one-dimensional convolutional operation with kernel size $\:K$ is applied. The outputs are then passed through a sigmoid to generate normalized channel-wise attention maps:

4 $$\:{s}^{GAP}=\:\sigma\:\left(Conv1D\left({z}^{GAP},K\right)\right)$$

5 $$\:{s}^{GMP}=\:\sigma\:\left(Conv1D\left({z}^{GMP},K\right)\right)$$

Subsequently, the two attention maps are fused through an element-wise maximum operation, allowing the module to emphasize the most informative spectral regions captured by either pooling operation:

6 $$\:s=\text{m}\text{a}\text{x}({s}^{GAP},\:{s}^{GMP})$$

Finally, the fused attention map is applied multiplicatively to the original feature map to recalibrate the channel responses, thereby dynamically enhancing critical wavelengths while suppressing less informative ones:

7 $$\:{\stackrel{\sim}{x}}_{k}\left(i\right)=\:{s}_{k}\times\:{x}_{k}\left(i\right),\:k=1,\:.\:.\:.\:.\:,C;i=1,\:.\:.\:.\:.\:,m$$

where, $\:\sigma\:(\bullet\:)$ denotes the sigmoid activation function, $\:C$ denotes the number of channels and $\:m$ represents the number of spectral features per channel, and $\:{x}_{k}\left(i\right)$ is the $\:i$-th spectral feature in the $\:k$-th channel. Note that while the original spectral input is a single-channel signal with 331 spectral variables, the term “channel” here refers to the learned feature map channels generated by intermediate convolutional layers of the network.

#### Spectral residual gate (SRG) module

**Fig. 4:**
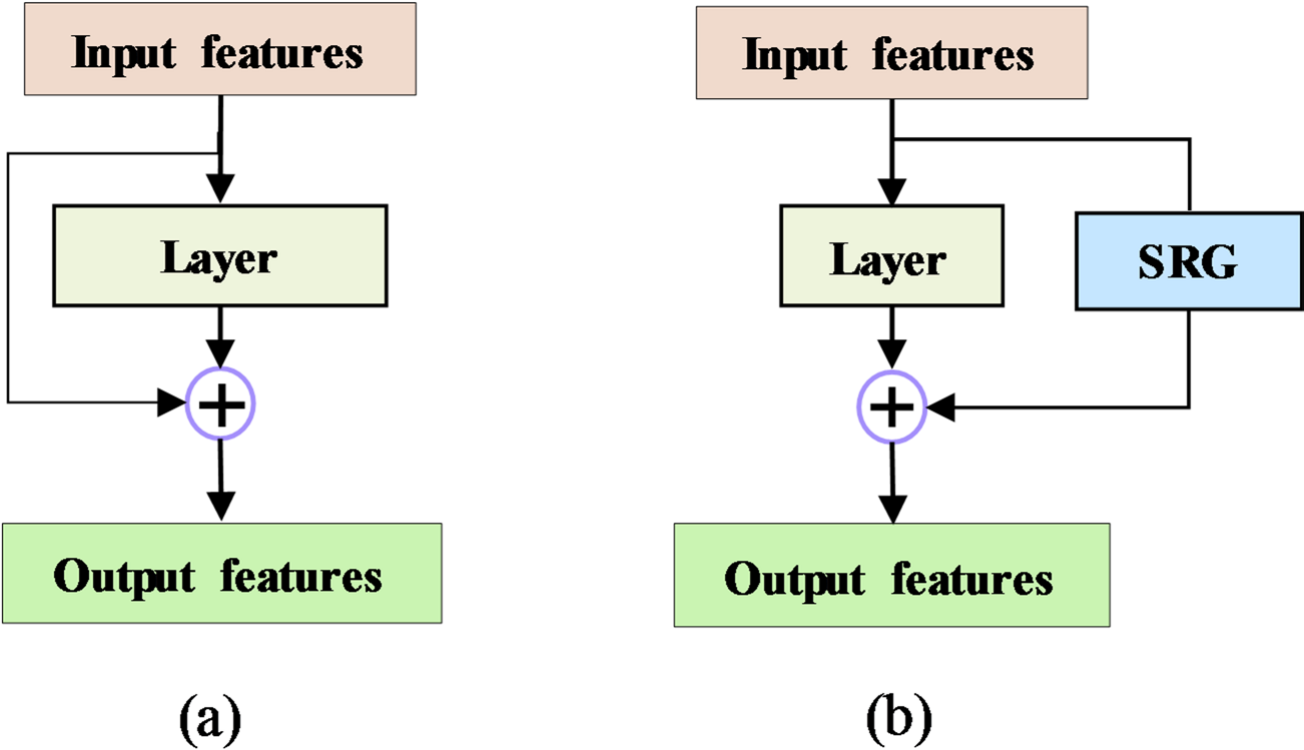
(**a**) Conventional residual connection, (**b**) SRG module.

Residual connections^48^ have been widely used in deep neural networks to mitigate the degradation problem, wherein deeper networks tend to suffer from vanishing gradients^49^. Traditional residual connections, however, simply add earlier layer outputs directly to the current feature map (Fig. 4a), which can overwhelm important information when gradient issues are not prevalent. In spectral analysis, neighboring wavelengths are highly correlated, resulting in smooth spectral patterns. However, crucial information often resides in subtle local variations or distortions. Standard residual connections may inadvertently introduce noise or redundancy in such contexts by treating all residual information equally. Inspired by^42^, SRG module is introduced to address this limitation by adaptively controlling the residual contribution based on the current feature representation (Fig. 4b). The SRG module helps preserve fine spectral structures while maintaining overall smoothness, enabling the network to focus on meaningful variations critical for accurate spectral analysis. Formally, given an input feature $\:x\varepsilon {\mathbb{R}^{C \times \:m}}$, and residual feature $\:r{\epsilon\mathbb{R}}^{d}$, the SRG module computes the adaptive residual addition as:

8 $$\:\widehat{x}=x+\:\alpha\:\cdot\:{\sigma\:(W}_{3}({W}_{2}\cdot\:Act({W}_{1}\cdot\:Conct(x,r)\left)\right))\cdot\:r$$

where, $\:Conct\left(x,r\right)$ denotes the concatenation of the input $\:x$ and residual$\:\:r$, $\:{W}_{1}{\epsilon\mathbb{R}}^{2d\times\:{h}_{d}}$, $\:{W}_{2}{\epsilon\mathbb{R}}^{{h}_{d}\times\:d}$ and $\:{W}_{3}{\epsilon\mathbb{R}}^{d\times\:1}$ represents learned weight matrices, $\:Act\left(\cdot\:\right)$ indicates non-linear activation function, $\:\sigma\:(\bullet\:)$ denotes sigmoid function, and $\:\alpha\:$ is learnable scalar initialized to 1. This formulation allows the SRG module to adaptively modulate residual connections, enabling the network to emphasize important spectral patterns while minimizing noise.

#### Deconvolution layer (Conv1dTranspose)

Deconvolution layers (also known as transposed convolution layers) are commonly used to reconstruct higher-dimensional representations from compressed feature maps by performing up-sampling^47^, while simultaneously learning to preserve and restore critical features. Their operation is mathematically analogous to the inverse of a standard convolution and can be expressed as:

9 $$\:y\left[i\right]=\:\sum\:_{j=0}^{k-1}w\left[j\right]\cdot\:x\left[i-j\cdot\:s\right]+b$$

Under same padding, the resulting output sequence expressed as: $\:Y={y}_{1},{y}_{2},.\:.\:.\:.\:.\:{y}_{n}$. Following each transposed convolution layer, BN is applied then a non-linear activation function is used to enhance the model’s capacity to reconstruct complex spectral structures and subtle signal variations.

Flattened and dense layer: The flattened layer serves as a bridge between the spectral encoder and the classification stages by converting multi-channel feature maps into a one-dimensional vector. This transformation ensures that the spectral patterns are preserved for subsequent processing. The dense layer then maps this high-dimensional vector into a fixed-length output, capturing the complex relationships across the spectral dimensions. In the SpecFuseNet architecture, the final dense layer, equipped with a Softmax activation function, produces class probability scores, translating the extracted features into precise predictions based on subtle wavelength variations.

#### Optimizer

The optimization strategy plays a critical role in training deep learning models effectively. Among the widely used optimizers; Stochastic Gradient Descent (SGD) updates model parameters by computing the gradient of the loss function with respect to each parameter and adjusting the parameters in the direction that minimizes the loss^50^. The update rule is given by.

10 $$\:{\theta\:}_{t+1}={\theta\:}_{t}-\alpha\:\cdot\:{g}_{t},\:{g}_{t}=\:{\nabla\:}_{\theta\:}J\left({\theta\:}_{t}\right)$$

Here, $\:{\theta\:}_{t}$ represents the model parameters at iteration $\:t$, $\:\alpha\:$ is the learning rate, and $\:{g}_{t}$ is the gradient of the loss function $\:J\left(\theta\:\right)$ evaluated at $\:{\theta\:}_{t}$. Adadelta improves upon Adagrad by using a decaying average of squared gradients to address diminishing learning rates^51^. The update rule is:

11 $$\:{\theta\:}_{t+1}=\:{\theta\:}_{t}+\:\frac{\varDelta\:{\theta\:}_{t}}{\sqrt{E{\left[{g}^{2}\right]}_{t}+\in\:}}$$

where, $\:E{\left[{g}^{2}\right]}_{t}$ is the exponentially weighted average of squared gradients, and $\:\in\:$ is a small constant for numerical stability. RMSprop improves training stability by using a moving average of squared gradients to adjust the learning rate^52^. The update rule is:

12 $$\:{v}_{t}=\:\beta\:\cdot\:{v}_{t-1}+(1-\beta\:)\cdot\:{g}_{t}^{2}$$

13 $$\:{\theta\:}_{t+1}=\:{\theta\:}_{t}+\:\frac{\alpha\:{\cdot\:g}_{t}}{\sqrt{{v}_{t}+\in\:}}$$

In this equation, $\:{v}_{t}$ denotes the moving average of squared gradients, and $\:\beta\:$ is the decay rate (set to 0.99). Adaptive Moment Estimation (Adam) combines momentum and adaptive learning rates by maintaining moving averages of both gradients and squared gradients, with bias correction^53^. The update rule is:

14 $$\:{m}_{t}=\:{\beta\:}_{1}\cdot\:{m}_{t-1}+(1-{\beta\:}_{1})\cdot\:{g}_{t}$$

15 $$\:{v}_{t}=\:{\beta\:}_{2}\cdot\:{v}_{t-1}+(1-{\beta\:}_{2})\cdot\:{g}_{t}^{2}$$

16 $$\:{\widehat{m}}_{t}=\frac{{m}_{t}}{1-{\beta\:}_{1}^{t}},\:{\widehat{v}}_{t}=\frac{{v}_{t}}{1-{\beta\:}_{2}^{t}}$$

17 $$\:{\theta\:}_{t+1}=\:{\theta\:}_{t}-\:\frac{\alpha\:\cdot\:{\widehat{m}}_{t}}{\sqrt{{\widehat{v}}_{t}}+\in\:}$$

where, $\:{m}_{t}$ and $\:{v}_{t}$ are the first and second moment estimates, $\:{\beta\:}_{1}$ and $\:{\beta\:}_{2}$ are exponentially decay rates (0.9 and 0.999, respectively), and $\:{\widehat{m}}_{t}$ and $\:{\widehat{v}}_{t}$ are bias-corrected moment estimates. By adapting the learning rate for each parameter during training, the Adam optimizer optimizes both computational efficiency and performance, minimizing complexity while ensuring effective model training.

Activation function: Activation functions introduce nonlinearity to the model, enabling it to fit complex, nonlinear data. Without activation functions, a network can only perform linear mappings, limiting its ability to model intricate relationships. Therefore, activation functions are crucial for enhancing the network’s fitting capability. The commonly used activation functions are listed in Table 1.

**Table 1:** List of activation function used.

Activation function	Equation
ELU (Exponential Linear Unit)	$$\:\text{f}\left(\text{x}\right)=\left\{\begin{array}{c}x,\:\:x\ge\:0\\\:\alpha\:*({\text{e}}^{\text{x}}-1),\:\:x<0\end{array}\right.$$
ReLU (Rectified Linear Unit)	$$\:\text{f}\left(\text{x}\right)=\left\{\begin{array}{c}x,\:\:x\ge\:0\\\:0,\:\:x<0\end{array}\right.$$
LeakyReLU	$$\:\text{f}\left(\text{x}\right)=\left\{\begin{array}{c}x,\:\:x\ge\:0\\\:\alpha\:*x,\:\:x<0\end{array}\right.$$
PReLU (Parametric ReLU)	$$\:\text{f}\left(\text{x}\right)=\left\{\begin{array}{c}x,\:\:x\ge\:0\\\:\alpha\:*x,\:\:x<0\end{array}\right.$$

In LeakyReLU, $\:\alpha\:$ is a fixed small constant (0.01), and In PReLU, $\:\alpha\:$ is learnable parameter during training.

Loss functions and regularization: The proposed model employs a combined loss function with equal contributions$\:\:\left(50\%\right)\:$from training and classification losses. This dual-loss setup forms the basis of the hybrid learning strategy, which enables the network to simultaneously optimize for accurate classification and faithful spectral reconstruction. Such a joint objective enhances feature learning and promotes better generalization. The training loss is based on mean squared error (MSE), which quantifies the difference between the original and reconstructed spectral data:

18 $$\:{L}_{MSE}=\frac{1}{m}\:\sum\:_{i=1}^{m}{({y}_{i}-{\widehat{y}}_{i})}^{2}\:$$

Here, $\:{y}_{i}$ and $\:{\widehat{y}}_{i}$ represents the original and reconstructed spectral values, and $\:m$ denotes sample size. For classification, the model uses sparse categorical cross-entropy to evaluate the divergence between the true and predicted class distributions:

19 $$\:{L}_{CE}=-\frac{1}{N}\sum\:_{i=0}^{N-1}\sum\:_{k=0}^{K-1}{y}_{i,k}\text{log}{(p}_{i,k})$$

where, $\:{y}_{i,k}$ the actual class of $\:i$th spectrum samples of $\:k$, with a total class $\:K$ and $\:N$ samples, and $\:{p}_{i,k}$ denotes predicted probability of $\:i$-th spectrum sample at $\:k$-th class value. This loss is especially effective in handling overlapping class boundaries in spectral data, where spectral features may be similar across classes. In this study, the classification targets include 24 barley, 19 chickpea, and 10 sorghum varieties, with the sample size defined by the respective dataset.

$\:L2$ regularization (weight decay) is used to reduce overfitting by penalizing large weights and improving generalization of model on unseen spectral data. It encourages smoother weight values without forcing them to zero. The total loss with $\:L2$ regularization expressed as:

20 $$\:{L}_{total}=\:{w}_{1}\cdot\:{L}_{1}+{w}_{2}\cdot\:{L}_{2}+\:\lambda\:\sum\:{w}_{i}^{2}$$

where, $\:{L}_{1}$ is the MSE loss function, $\:{L}_{2}$ is cross-entropy loss function, $\:{w}_{1}$ and $\:{w}_{2}$ are their respective weights, $\:\lambda\:$ denotes regularization coefficient (set 0.01), and $\:{w}_{i}$ indicates the weight of convolutional layers.

**Evaluation indices of the model**: The evaluation of grain variety classification based on spectral data utilizes metrics includes Accuracy (Acc), Precision (Pre), Recall (Re), F1-score (F1), and confusion matrix. Accuracy measures the overall correctness of the model, particularly when the class distribution is balanced. Precision evaluates the proportion of correctly identified positive instances, while Recall reflects the model’s ability to detect all relevant positive cases, which is critical when reducing false negatives is a priority. The F1-score provides a balanced assessment when both Precision and Recall are important. The confusion matrix offers a detailed comparison between predicted and actual class labels. These metrics, when considered as a whole, offer a uniform framework for evaluating and contrasting various models, thereby facilitating the discernment of the most efficacious model for the classification of grain varieties utilizing spectral characteristics.

**Experimental setup**: The implementation and optimization of the deep learning model were executed utilizing Python (3.9) alongside the open-source deep learning framework TensorFlow/Keras (2.10.0)^54^. The experimental procedures were carried out on a desktop workstation that was outfitted with an NVIDIA GeForce RTX 4060 GPU, an Intel^®^ Core™ i7-14700 F CPU operating at 2.10 GHz, 16 GB of RAM, and was functioning on the Microsoft Windows 11 operating system.

## Results and discussion

This section presents experiments conducted on the NIR spectral datasets of grain varieties. The results are analyzed to highlight the performance of the proposed SpecFuseNet model compared to alternative configurations. To assess the model’s robustness and generalization capability, five-fold stratified cross-validation was applied to three grain variety datasets: barley, chickpea, and sorghum. Considering the critical impact of the optimizer and activation function on neural network performance, a comprehensive set of experiments was performed. Each dataset was evaluated using four optimizers: Adam, RMSprop, SGD, and Adadelta in combination with four activation functions: ELU, ReLU, LeakyReLU, and PReLU. This approach was intended to assess the model’s adaptability and consistency across different optimization strategies and grain types. Training stability and convergence were further enhanced using an adaptive learning rate scheduler. The initial learning rate was set to 0.0001, and the scheduler automatically reduced it by a factor of 0.5 if the validation loss did not improve for five consecutive epochs. This strategy helped mitigate overshooting, facilitated fine-tuning during later training stages, and contributed to improved model performance. The parameter configuration of the SpecFuseNet model for feature extraction and classification is provided in Table 2.

### Barley variety classification

The classification results for the barley spectral dataset, using the proposed SpecFuseNet, are summarized in Table 3. The combination of the Adam optimizer and the ELU activation function achieved the highest performance, with a test accuracy 89.72%, precision 90.19%, recall 89.72%, and an F1-score 89.56%. These high precision, recall, and F1-score values indicate that SpecFuseNet achieved highly reliable classification, accurately identifying most barley varieties while minimizing both false positives and false negatives. Among the different optimizer–activation function combinations, Adam paired with ELU consistently outperformed all others across all evaluation metrics. This strong performance can be attributed to ELU’s smooth, non-linear activation characteristics, which effectively capture the variability in barley, and the adaptive learning capabilities of the Adam optimizer, which enhanced training stability and convergence. RMSprop and SGD with ELU activation also delivered competitive performances, achieving test accuracies of 87.5% and 86.11%, respectively. In contrast, Adadelta exhibited lower results, with a maximum test accuracy of 71.39% when combined with ELU. Overall, these findings demonstrate that the Adam-ELU configuration provided the most stable and accurate performance for barley spectral classification using SpecFuseNet.

**Table 2:** SpecFuseNet NIR spectral feature extraction and classification network parameter configuration.

Parameters
**Filters** Filter_1 = 32, Filter_2 = 16, Filter_3 = 8, Filter_out = 1, k = 3, k_regularizer = l2(0.01)
**Spectral Encoder** Filter_1 to Filter_3 Conv1D: (k_size = k, padding = ‘same’, k_regularizer), BN, Activation = ‘elu’ Fused ECA(k_size = 5) SRG (hidden_dim = 92, alpha = learnable scalar)
**Spectral Decoder** Filter_3 to Filter_1 Conv1DTranspose: (k_size = k, padding = ‘same’, k_regularizer), BN, Activation = ‘elu’ Fused ECA (k_size = 5) SRG (hidden_dim = 92, alpha = learnable scalar) Conv1DTranspose: (Filter_out, k_size = k, padding =’ same’), BN, Activation = ‘elu’
**Spectral Classifier** Flatten() Dense: (128, activation = ELU, k_regularizer) Dense: (num_classes, activation = ‘softmax’)
**Training** cross-validation: Stratified 5-fold cross-validation optimizer: Adam (learning_rate = 0.0001) with learning rate scheduler losses: [‘mse’, ‘sparse_categorical_crossentropy’], loss_weights = [0.5, 0.5] metrics: [‘accuracy’] epochs: 300 batch_size: 32

**Table 3:** Feature extraction and classification performance of specfusenet using different optimizers and activation functions on the barley spectral dataset.

Optimizer	Activation function	Acc (%)	Pre (%)	Rec (%)	F1 (%)
Adadelta	ReLU	57.50	56.76	57.50	54.00
	PReLU	58.33	57.43	58.34	55.64
	LeakyReLU	66.11	65.72	66.11	64.01
	ELU	71.39	69.45	71.39	69.34
SGD	ReLU	82.50	82.77	82.50	82.14
	PReLU	84.17	84.37	84.17	83.91
	LeakyReLU	86.67	87.72	86.67	86.62
	ELU	86.11	86.56	86.11	85.91
RMSprop	ReLU	82.78	82.74	82.78	82.21
	PReLU	85.83	86.08	85.83	85.63
	LeakyReLU	86.94	87.41	86.94	87.00
	ELU	87.50	88.12	87.50	87.54
Adam	ReLU	85.56	86.37	85.56	85.34
	PReLU	86.67	87.03	86.67	86.46
	LeakyReLU	87.22	87.64	87.22	87.24
	ELU	**89.72**	**90.19**	**89.72**	**89.56**

**Fig. 5:**
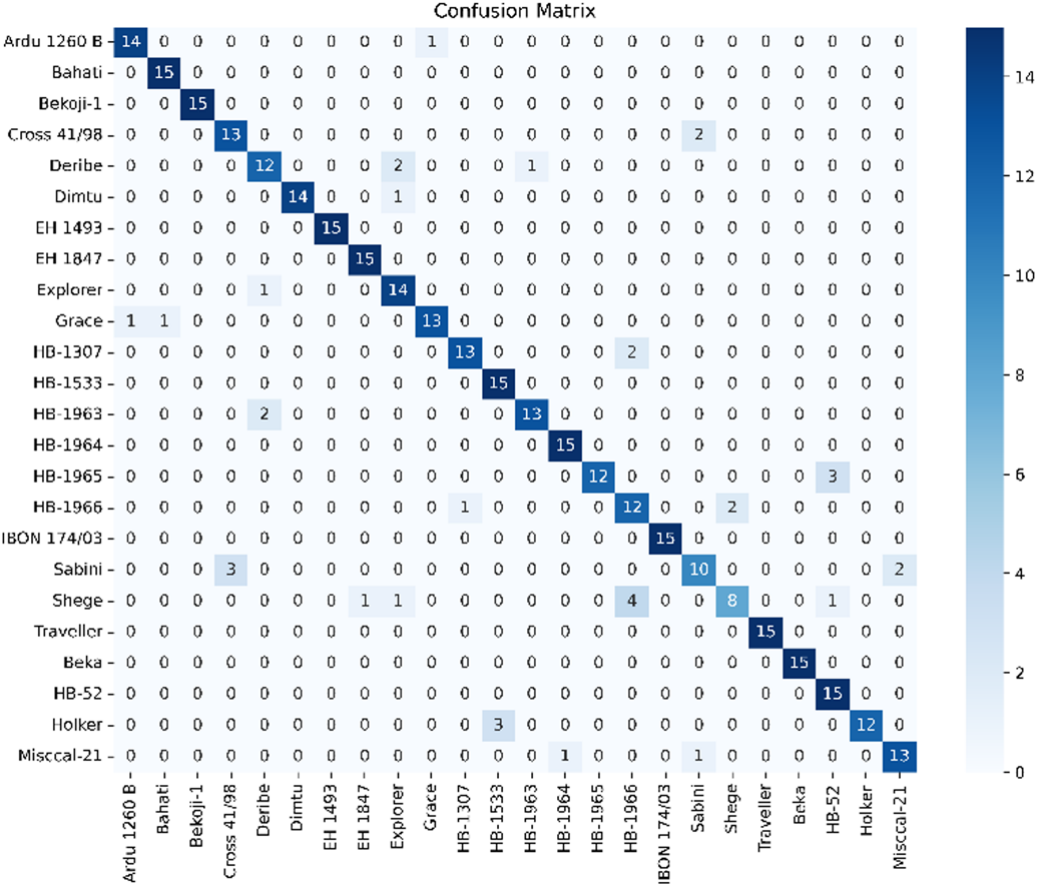
Confusion matrix of barley variety classification with an overall accuracy of 89.72%.

As shown in Fig. 5, SpecFuseNet achieved an overall accuracy of 89.72% on the barley dataset, with ten varieties classified perfectly and the rest exceeding 80% accuracy. However, notable misclassifications occurred with the Sabini and Shege varieties. Sabini was often confused with Cross 41/98 and Misccal-21, likely due to similarities in seed morphology and moisture content affecting light scattering and absorption. Shege was frequently misidentified as HB-1966, EH 1847, Explorer, or HB-52, probably because of overlapping chemical compositions like protein and carbohydrate content. These errors underscore the difficulty of distinguishing cultivars with closely aligned NIR spectral profiles, especially when spectral variability is limited or affected by environmental factors during sampling. Nonetheless, SpecFuseNet’s use of FusedECA and SRG modules helped reduce feature redundancy and enhance class-specific spectral focus, boosting classification robustness.

### Chickpea variety classification

Table 4 summarizes the classification results for chickpea varieties, achieved using the SpecFuseNet model. The Adam optimizer combined with the ELU activation function achieved the highest performance, with a test accuracy of 96.14%. The precision (96.45%) indicates the model’s high accuracy in identifying the target class, while recall (96.14%) reflects the proportion of true instances correctly classified. 96.10% F1-score reflects a balanced trade-off between precision and recall, indicating the model’s strong overall performance by effectively accounting for both false positives and false negatives. This strong performance is attributed to ELU’s non-linear activation, which captures variability in chickpea morphology, and Adam’s adaptive learning, which enhances training stability and convergence. A comparative analysis of different optimizers and activation functions reveals that ELU consistently performed well across all optimizers, particularly when paired with Adam and RMSprop, achieving test accuracies of 96.14% and 95.44%, respectively. ReLU, LeakyReLU, and PReLU configurations yielded slightly lower metrics, while Adadelta showed the weakest performance, with a maximum test accuracy of 80.35%, confirming its limited effectiveness for chickpea spectral classification.

**Table 4:** Feature extraction and classification performance of specfusenet using different optimizers and activation functions on the Chickpea spectral dataset.

Optimizer	Activation function	Acc (%)	Pre (%)	Rec (%)	F1 (%)
Adadelta	ReLU	70.87	74.04	70.75	70.51
	PReLU	74.74	77.20	74.64	74.74
	LeakyReLU	75.09	76.87	74.93	74.23
	ELU	80.35	82.79	80.27	79.93
SGD	ReLU	91.22	91.93	91.22	91.08
	PReLU	91.58	92.10	91.55	91.47
	LeakyReLU	92.98	93.39	92.98	92.88
	ELU	93.33	93.50	93.33	93.23
RMSprop	ReLU	92.98	93.39	92.98	92.68
	PReLU	93.68	94.28	93.65	93.72
	LeakyReLU	95.08	95.60	95.08	95.05
	ELU	95.44	95.76	95.44	95.40
Adam	ReLU	94.03	94.44	94.03	94.04
	PReLU	94.38	94.63	94.38	94.38
	LeakyReLU	95.08	95.20	95.08	94.82
	ELU	**96.14**	**96.45**	**96.14**	**96.10**

**Fig. 6:**
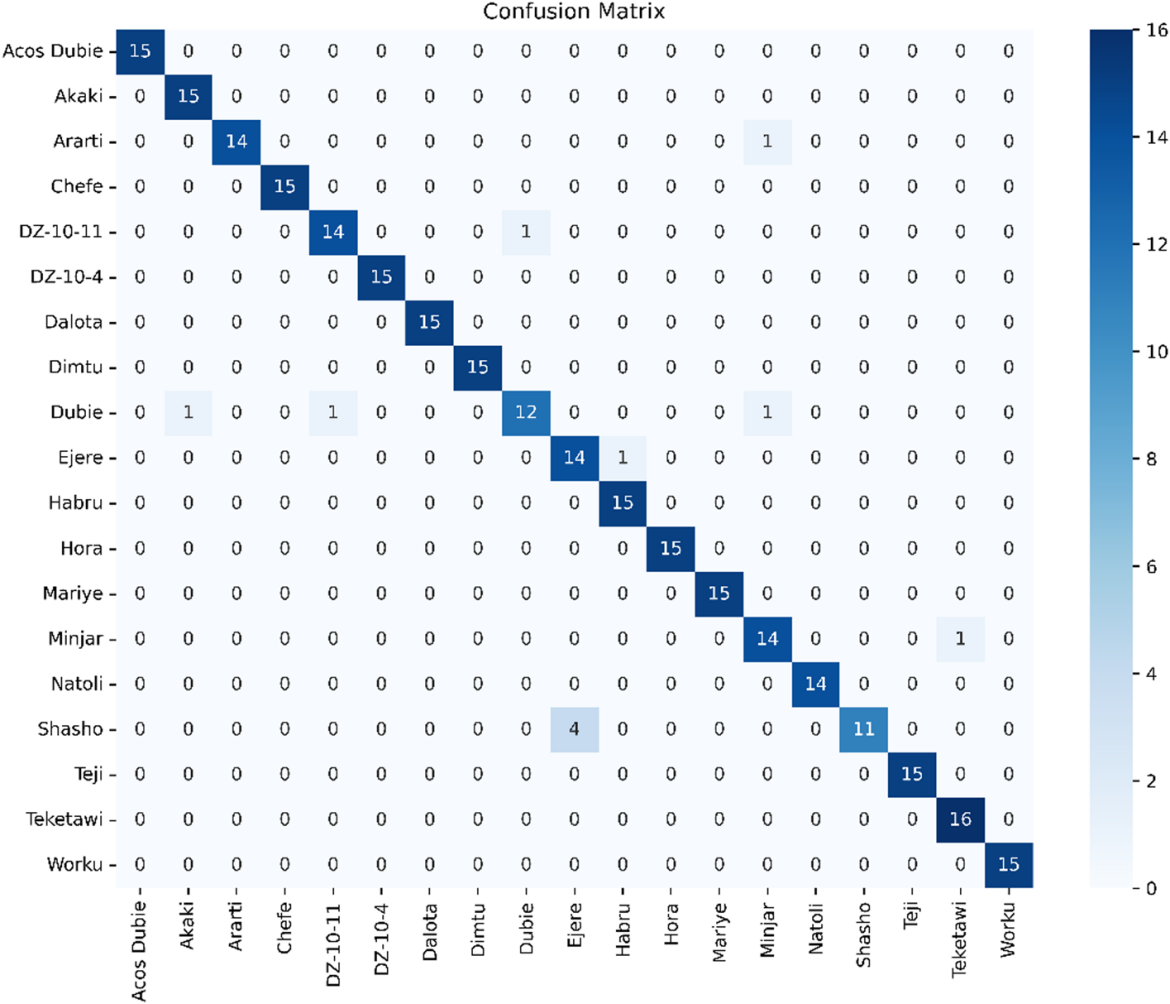
Confusion matrix of chickpea variety classification with an overall accuracy of 96.14%.

In Fig. 6, SpecFuseNet achieved an impressive accuracy of 96.14% on the chickpea dataset, with most varieties showing excellent classification performance. The primary misclassification involved the Shasho variety, which was often predicted as Ejere or Teketawi. This confusion likely stems from similarities in cotyledon density and seed coat thickness, which affect reflectance patterns. Moreover, these varieties may share agronomic lineage or undergo similar post-harvest treatments like drying, resulting in nearly identical NIR spectral signatures. Such spectral overlap highlights the challenge of distinguishing varieties within species that possess uniform biochemical traits, especially when using only raw NIR spectral data. While the ELU-activated SpecFuseNet model mitigates these issues through nonlinear feature representation, subtle biochemical similarities can still lead to occasional classification confusion.

### Sorghum variety classification

We evaluated SpecFuseNet on the sorghum spectral dataset, which showed in Table 5. The Adam optimizer combined with the ELU activation function achieved the highest test accuracy of 90.67%. 91.23% precision indicates the model’s high accuracy in identifying the target class, while 90.65% recall reflects the proportion of true instances correctly classified. A 90.65% F1-score balances precision and recall, providing an overall measure of the model’s performance by accounting for both false positives and false negatives. A comparative analysis of different optimizers and activation functions reveals that ELU consistently performed well across all optimizers, particularly when paired with Adam, achieving a test accuracy of 90.67%. RMSprop with ELU also performed well, with a test accuracy of 88.67%. In contrast, models using ReLU, LeakyReLU, or PReLU showed slightly lower classification metrics. Adadelta, however, showed the weakest performance, with a maximum test accuracy of 78%, confirming its limited effectiveness for sorghum spectral classification.

**Fig. 7:**
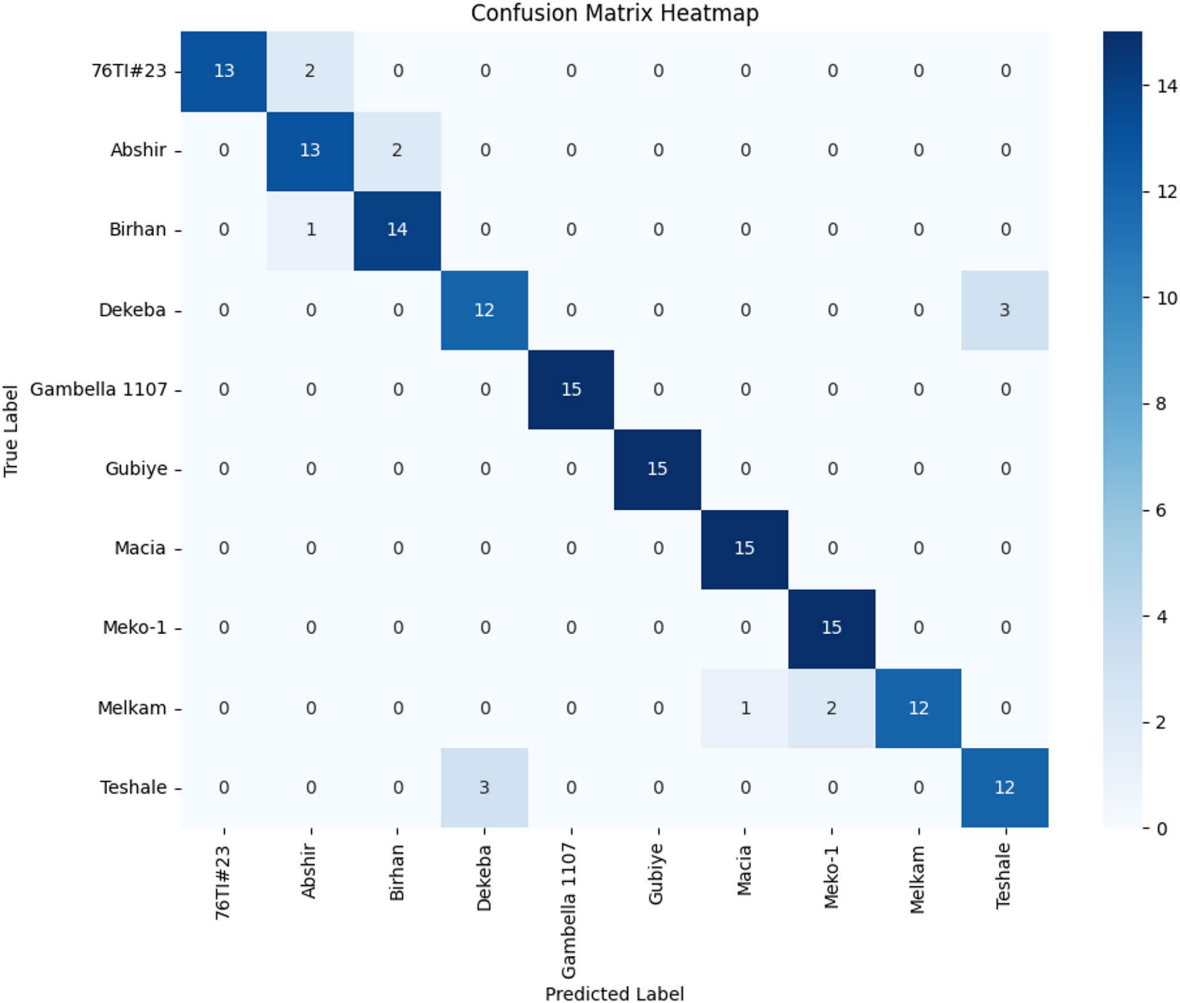
Confusion matrix of sorghum variety classification with an overall accuracy of 90.67%.

According to Fig. 7, SpecFuseNet achieved a classification accuracy of 90.67% on the sorghum dataset, with four varieties classified perfectly and most others exceeding 85% accuracy. Misclassifications occurred mainly in three varieties, with accuracies closer to 80%, likely due to overlapping starch and lignin content. Additionally, sorghum varieties often share similar kernel structures and endosperm types, leading to converging spectral patterns. Grain surface characteristics, such as roughness and coloration, can also influence light penetration and reflectance, particularly when using compact portable NIR devices like the SCIO scanner. Despite these challenges, SpecFuseNet maintained strong performance, demonstrating that its combined residual and attention mechanisms effectively suppress irrelevant spectral noise while highlighting chemically meaningful differences.

**Table 5:** Feature extraction and classification performance of specfusenet using different optimizers and activation functions on the sorghum spectral dataset.

Optimizer	Activation function	Acc (%)	Pre (%)	Rec (%)	F1 (%)
Adadelta	ReLU	73.33	74.52	73.34	70.20
	PReLU	72.00	76.13	72.00	67.89
	LeakyReLU	78.67	79.99	78.67	76.61
	ELU	78.00	77.62	78.00	76.68
SGD	ReLU	83.34	84.08	83.34	82.68
	PReLU	85.34	85.71	85.33	84.99
	LeakyReLU	86.67	87.49	68.66	86.39
	ELU	86.00	87.76	86.00	85.68
RMSprop	ReLU	82.67	82.75	82.68	82.27
	PReLU	86.00	86.75	86.00	85.94
	LeakyReLU	87.33	88.14	87.34	87.23
	ELU	88.67	88.87	88.67	88.58
Adam	ReLU	86.00	86.61	86.00	84.41
	PReLU	86.67	87.99	86.66	86.31
	LeakyReLU	88.67	89.24	88.67	88.37
	ELU	**90.67**	**91.23**	**90.65**	**90.50**

Across all datasets, the combination of the Adam optimizer with the ELU activation function consistently yielded superior performance in both feature extraction and classification. This outcome highlights the benefits of ELU’s non-zero gradient, which facilitates capturing the complexity and subtle variations within spectral features. Meanwhile, Adam’s adaptive learning capabilities enhance convergence and training stability, leading to more effective optimization. The observed misclassification patterns generally corresponded to known spectral similarities among varieties, indicating the model’s sensitivity to nuanced biochemical differences in the NIR spectra. Such misclassifications are expected when varieties exhibit overlapping spectral characteristics, underscoring the critical role of precise feature extraction.

In summary, SpecFuseNet combined with the Adam-ELU configuration delivers excellent performance in NIR spectral data classification. Its feature extraction is strengthened by the FusedECA module, which enhances channel-wise feature selection, while the SRG module effectively controls spectral attention, guiding the model to focus on the most informative features. Together, these components enable robust representation learning and improved class separation, even when differentiating spectrally similar varieties.

### Feature space visualization

**Fig. 8:**
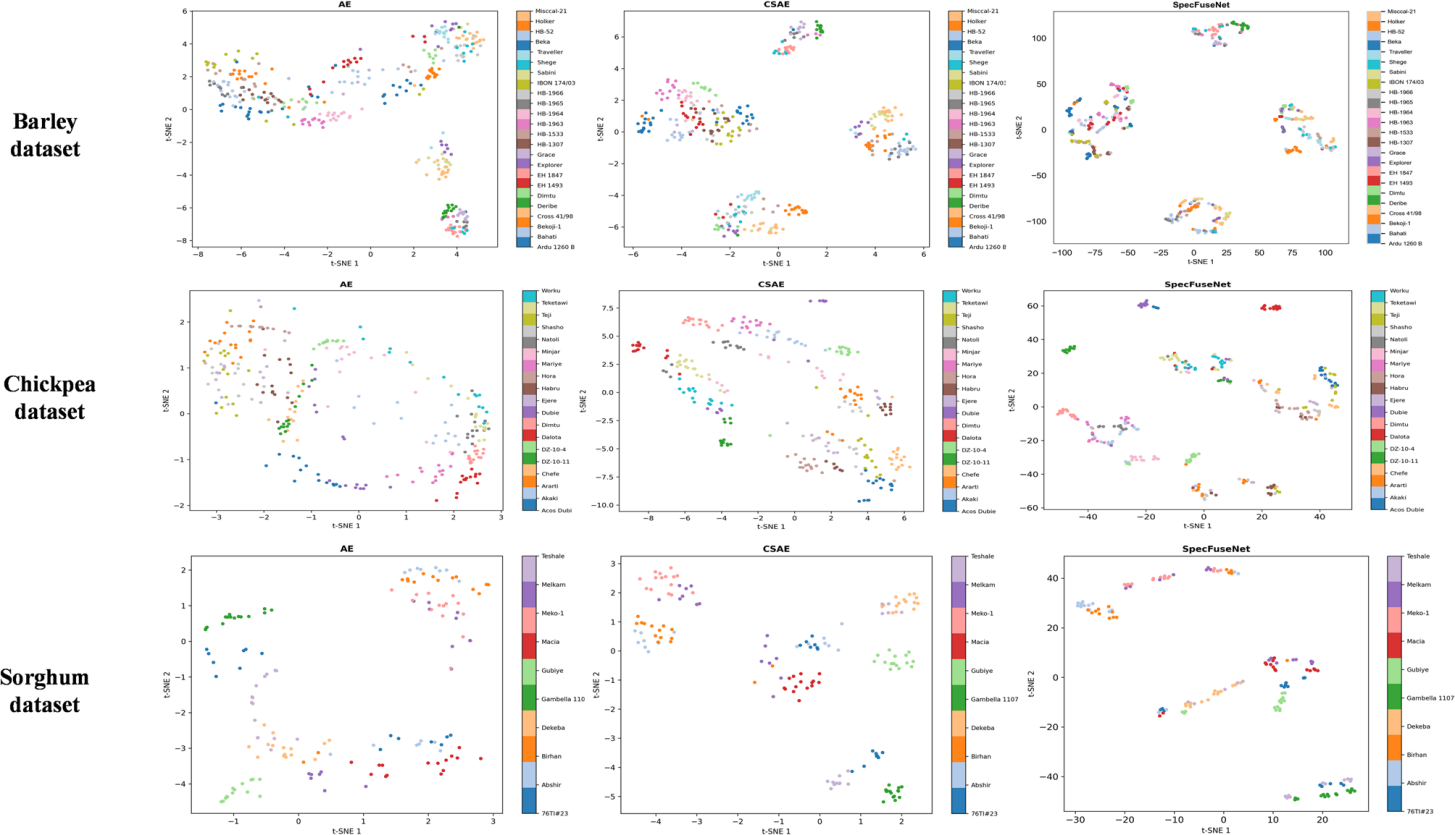
Feature space visualization using t-SNE of latent features learned by AE, CSAE, and SpecFuseNet on NIR spectral datasets of barley, chickpea, and sorghum.

To qualitatively assess the discriminative capacity of learned features, we visualized the latent representations of AE, CSAE, and SpecFuseNet using t-Distributed Stochastic Neighbor Embedding (t-SNE). As shown in Fig. 8, SpecFuseNet forms more compact and clearly separated clusters across the barley (24 varieties), chickpea (19 varieties), and sorghum (10 varieties) datasets. In contrast, AE and CSAE show dispersed and overlapping clusters, reflecting weaker class separability. These results demonstrate that SpecFuseNet learns more discriminative and structured spectral embeddings, further validating the effectiveness of its FusedECA and SRG modules for robust feature extraction.

### Conventional feature extraction and classification methods

To reduce the dimensionality of high-dimensional spectral data, PCA projects the original input data into a lower-dimensional space that maximizes variance, simplifies model computation, and preserves essential information^55^. The PCA process for spectral feature extraction can be described as follows:

Given a dataset $\:X\in\:{\mathbb{R}}^{n\times\:d}$, where $\:n$ is the number of spectrum data and $\:d$ is the number of original features:

i. Center the Data: Given a dataset $\:X\in\:{\mathbb{R}}^{n\times\:d}$, the data is centered by subtracting the mean of each feature:

21 $$\:{X}_{centered}=X-\mu\:$$

Here, $\:n$ is spectral sample number and $\:d$ is original features number, $\:{X}_{centered}$ is centralized spectrum data, $\:X$ denotes original spectrum data, and $\:\mu\:$ for mean value.

ii. Compute the covariance matrix ($\:C$) on the spectrum data:

22 $$\:C=\:\frac{1}{n-1}{{X}_{centered}}^{T}{X}_{centered}$$

where, $\:{{X}_{centered}}^{T}$ represents transpose of the $\:{X}_{centered}$ matrix.

iii. Calculate eigenvalues and eigenvectors of $\:C$:

23 $$\:C\:=\:V\lambda\:{V}^{T}$$

where, $\:V$ is the matrix of feature vectors and $\:\lambda\:$ is the diagonal matrix containing the eigenvalues which the variance captured by each corresponding eigenvector. The eigenvectors corresponding to the top $\:k$ largest eigenvalues are selected to form the matrix $\:{V}_{k}$ which represents the principal components of the reduced feature space. Project the data onto the new space:

24 $$\:Z=\:{X}_{centered}{V}_{k}$$

where, $\:Z$ is the reduced-dimensional feature matrix $\:Z\in\:{\mathbb{R}}^{n\times\:k}$.

The reduced spectral data, obtained through PCA, is then used with traditional classification algorithms such as SVM, RF, and XGBoost. The effectiveness of the PCA-based features is evaluated using the NIR spectral dataset of grain varieties (barley, chickpea, and sorghum) under stratified 5-fold cross-validation to ensure balanced class distributions across folds. The parameter settings for traditional classifiers are as follows: For SVM, a linear kernel was used with $\:C=0.1$, a maximum of $\:200$ iterations, and class weight set to *‘balanced’*. Additionally, an SVM with an RBF kernel was configured with $\:C=1$, a maximum of $\:200$ iterations, and class weight set to *‘balanced’*. For the RF classifier, the number of estimators was set to $\:200$, with class weight also set to *‘balanced’*. In the case of XGBoost, the objective was specified as *‘multi: softmax’*, with $\:200\:$estimators and a learning rate of $\:0.1$.

The results of PCA classification for the barley, chickpea, and sorghum datasets show that PCA with linear SVM and RF consistently outperforms other classifiers. For barley, PCA with SVM (linear) achieved an accuracy of 82.78%, while PCA with RF came close at 82.22%. PCA with XGBoost and RBF-SVM showed lower results. In the chickpea dataset, PCA with SVM (linear) performed best with an accuracy of 91.93%, followed by PCA with RF at 90.88%. XGBoost and RBF-SVM performed moderately. For sorghum, PCA with SVM (linear) again led with 85.33% accuracy, with PCA and RF achieving similar results. XGBoost and RBF-SVM exhibited slightly lower performance. PCA improved linear separability, allowing linear SVM to build more effective decision boundaries, while RF captured additional non-linear patterns and remained robust to noise. On the other hand, RBF-SVM tended to overfit local variations, and XGBoost offered slightly lower overall performance. Full details are provided in Table 6.

**Table 6:** Classification performance of PCA with different classifiers on barley, chickpea, and sorghum datasets.

Dataset	Method	Acc (%)	Pre (%)	Rec (%)	F1 (%)
Barley	PCA + SVM *(‘linear’*)	82.78	83.68	82.78	82.68
	PCA + SVM *(‘rbf’)*	71.11	69.52	71.11	68.31
	PCA + RF	82.22	82.25	82.23	80.63
	PCA + XGBoost	72.22	72.68	72.22	71.12
Chickpea	PCA + SVM *(‘linear’*)	91.93	92.02	91.93	91.86
	PCA + SVM *(‘rbf’)*	80.70	81.67	80.74	80.70
	PCA + RF	90.88	92.12	90.87	90.96
	PCA + XGBoost	84.91	85.72	84.91	84.91
Sorghum	PCA + SVM *(‘linear’*)	85.33	85.71	85.33	85.12
	PCA + SVM *(‘rbf’)*	78.00	77.78	78.00	76.17
	PCA + RF	84.00	85.14	84.00	82.50
	PCA + XGBoost	78.00	77.91	78.00	77.37

To evaluate the effectiveness of features derived from PCA, and SpecFuseNet-encoder for spectral classification, empirical experiments were conducted using features extracted by the SpecFuseNet-encoder with the same set of classifiers for comparison. The SpecFuseNet encoder combined with linear SVM consistently outperformed other models, achieving accuracy scores of 85.28% for barley, 93.33% for chickpea, and 86.67% for sorghum. RF and XGBoost also delivered competitive results, while RBF-SVM underperformed, as shown in Table 7. The strong performance of linear SVM with SpecFuseNet-encoder and RF highlights SpecFuseNet’s capability to enhance spectral feature extraction, leading to more efficient classification. In conclusion, features extracted by SpecFuseNet outperform those derived from PCA in spectral classification, particularly in terms of precision, recall, and F1-score.

**Table 7:** Spectral classification results using SpecFuseNet-Encoder features across different classifiers.

Dataset	Method	Acc (%)	Pre (%)	Rec (%)	F1(%)
**Barley**	Encoder + SVM (‘linear’)	85.28	86.10	85.28	85.16
	Encoder + SVM (‘rbf’)	74.44	74.27	74.45	72.01
	Encoder + RF	79.72	79.08	79.72	77.48
	Encoder + XGBoost	76.67	77.77	76.66	76.12
**Chickpea**	Encoder + SVM (‘linear’)	93.33	94.07	93.30	93.23
	Encoder + SVM (‘rbf’)	83.51	85.76	83.38	83.30
	Encoder + RF	86.67	87.63	86.68	86.82
	Encoder + XGBoost	85.96	86.92	85.91	85.98
**Sorghum**	Encoder + SVM (‘linear’)	86.67	86.61	86.66	86.29
	Encoder + SVM (‘rbf’)	80.67	81.71	80.66	78.44
	Encoder + RF	83.33	84.03	83.34	82.23
	Encoder + XGBoost	84.67	84.60	84.67	84.13

#### A classification method based on deep learning techniques

The performance of the proposed SpecFuseNet model was evaluated using deep learning techniques through AE and CSAE, for comparative analysis. The CSAE model applies L1 regularization to the convolutional layers within the encoder to promote sparsity in the learned weights and further enforces sparsity by incorporating an L1 penalty on the encoded activations as an additional term in the loss function. This dual regularization strategy enhances the model’s ability to learn compact feature representations while improving robustness to noise through enforced activation sparsity.

A comparative experimental study was conducted on deep learning-based classification using the same NIR spectral dataset consisting of three grain varieties: barley, chickpea, and sorghum. To ensure a fair comparison, similar parameters such as layer architecture for AE models, batch size, learning rate, optimizer configurations, number of training epochs, and k-fold cross-validation, were maintained across all models, as outlined in Table 2. The parameter settings of AE and CSAE model are detailed in Table S1 and Table S2, respectively.

The evaluation results of the spectral classification experiments, conducted using AE and CSAE models, are presented in Table 8. The analysis of AE reveals that its classification accuracy is relatively low, with 70.83% for barley, 82.46% for chickpea, and 76.00% for sorghum under the same parameters. This suggests that AE struggles to capture the necessary discriminative features for effective classification. Although AE is computationally efficient, it still exhibits a high rate of misclassification across different categories. In contrast, CSAE delivers much better results, achieving 83.61% for barley, 91.23% for chickpea, and 83.00% for sorghum. The proposed SpecFuseNet feature extraction and classification outperforms both AE and CSAE deep learning techniques for grain variety classification.

**Table 8:** Evaluation results of AE and CSAE models for spectral Classification.

Dataset	Model	Evaluation parameters			
		Acc (%)	Pre (%)	Rec (%)	F1 (%)
Barley	AE	70.83	71.62	70.83	70.51
	CSAE	83.61	84.17	83.61	82.83
Chickpea	AE	82.46	82.17	82.45	82.04
	CSAE	91.23	91.86	91.23	90.69
Sorghum	AE	76.00	78.82	76.00	72.81
	CSAE	86.00	86.35	86.00	85.99

### Ablation study

To demonstrate the effectiveness of the proposed SpecFuseNet model, an ablation study was conducted to evaluate the contribution of each module, CAE, ECA, FusedECA, and SRG on three grain datasets (Table 9). The study results, highlight the performance improvements observed as each component of the SpecFuseNet architecture was added. The baseline CAE model, responsible for latent feature extraction and denoising from NIR spectra, showed moderate performance across all datasets, emphasizing its role in capturing essential spectral characteristics. Adding the ECA module to the CAE backbone improved performance by emphasizing relevant spectral bands, increasing accuracy to 86.94% on barley, 94.04% on chickpea, and 86.67% on sorghum.

Replacing ECA with the FusedECA module, which combines global average and max pooling, further enhanced performance. This fusion allowed the model to capture both global trends and key spectral features, resulting in more discriminative representations. In barley, the F1-score improved from 84.86 to 88.80%, on chickpea, the F1-score improved from 92.92 to 94.82%, while for sorghum, it improved from 85.69 to 87.80%. FusedECA refined attention and further boosted feature extraction and classification. Integrating the SRG module alongside FusedECA consistently achieved the best results for barley, chickpea, and sorghum, improving classification accuracy by filtering redundant features and emphasizing class-discriminative information. SRG’s contribution was key in achieving a perfect F1-score of 89.56% on the barley dataset, 96.10% on chickpea, and 90.50% on sorghum. The ablation study highlights the complementary roles of the FusedECA and SRG modules in SpecFuseNet. The FusedECA refines spectral feature extraction by capturing global trends and key features, while the SRG focuses on class-discriminative residuals, enhancing classification. Together, they deliver superior NIR spectral feature extraction and grain variety classification, demonstrating SpecFuseNet’s effectiveness for spectral data analysis.

**Table 9:** The comparison of ablation study based on different modules.

Dataset	CAE	ECA	Fused-ECA	SRG	Acc (%)	Pre (%)	Re (%)	F1 (%)
**Barley**	**√**				85.00	85.20	85.00	84.86
	**√**	√			86.94	87.53	86.94	86.99
	**√**		√		88.89	89.3	88.89	88.8
	**√**		√	√	89.72	90.19	89.72	89.56
**Chickpea**	√				92.98	93.33	92.93	92.92
	√	√			94.04	94.44	94.03	94.08
	√		√		94.74	95.21	94.74	94.82
	√		√	√	96.14	96.45	96.14	96.1
**Sorghum**	√				86.00	86.48	86.00	85.69
	√	√			86.67	87.52	86.67	86.69
	√		√		88.00	88.30	88.00	87.80
	√		√	√	90.67	91.23	90.65	90.50

The experimental results confirm that the proposed SpecFuseNet model effectively classifies barley, chickpea, and sorghum grain varieties using small-scale NIR spectral data. Integrating CAE with FusedECA and SRG modules significantly improved feature extraction, leading to high classification accuracies of 89.72%, 96.14%, and 90.67%, respectively. The Adam optimizer combined with ELU activation consistently outperformed other configurations, demonstrating strong adaptability across different grain types. Compared to PCA-based features combined with traditional classifiers (SVM, RF, and XGBoost) and baseline models like AE and CSAE, SpecFuseNet delivered superior performance, particularly in preserving critical spectral information. Preprocessing strategies, including SG filtering and standardization, along with adaptive learning rate scheduling, enhanced model convergence and training stability. The ablation study further validated the contribution of each module, showing that the FusedECA mechanism improved global and local feature emphasis, while the SRG module dynamically modulated residual connections to focus on class-specific spectral variations. Although minor misclassifications occurred between spectrally similar varieties, the model exhibited robust generalization, indicating that SpecFuseNet is a promising and scalable approach for grain variety classification using compact NIR spectrometers.

## Conclusion

This study presents SpecFuseNet, a deep learning architecture that CAE with FusedECA and SRG modules for efficient spectral feature extraction and accurate grain variety classification using compact NIR spectroscopy. Experimental evaluations on barley, chickpea, and sorghum datasets demonstrate that SpecFuseNet consistently outperforms traditional feature extraction techniques paired with machine learning classifiers, as well as baseline deep learning models. The architecture improves spectral representation by dynamically highlighting discriminative features while reducing the influence of redundant or noisy information. This fusion of attention and residual learning enables robust and interpretable modeling of high-dimensional spectral data. Furthermore, the incorporation of adaptive learning strategies and effective preprocessing enhances the model’s generalization and stability across diverse grain types. SpecFuseNet’s modular and lightweight design also supports seamless integration into laboratory and field-based NIR spectroscopy systems, providing a scalable and cost-effective solution for precision agriculture. Overall, these findings highlight the practical value of SpecFuseNet as a reliable and efficient tool for non-destructive grain cultivar identification, contributing to data-driven decision-making in agricultural and food systems.

## Supplementary Information

Below is the link to the electronic supplementary material.

Supplementary Material 1

## Data Availability

The spectral data that support the findings of this study are publicly available at: https://github.com/ChengHao1963/nir-spectra-dataset.
