# Supplementary material for: Attention-enhanced residual autoencoder for NIR spectral feature extraction and classification of grain varieties
Source: Sci Rep. 2025 Sep 24;15:32750. doi: 10.1038/s41598-025-17676-w (PMC12460850; doi:10.1038/s41598-025-17676-w)
Supplement: Supplementary file 1 — Supplementary Material 1 [file 41598_2025_17676_MOESM1_ESM.docx]

**Attention-Enhanced Residual Autoencoder for NIR Spectral Feature Extraction and Classification of Grain Varieties**

**Table S1:** AE spectral feature extraction and classification network parameter configuration

| **Parameters** |
| --- |
| **Filters**  Units_1 = 32, Units_2 = 16, Units_3 = 8, Units_out = 8 |
| **Encoder:** Units_1 to Units_3  Flatten ()  Dense (Units_3, activation = ‘elu’) |
| **Decoder:** Units_2 to Units_1  Dense (np.prod(input_shape_data), activation = ‘sigmoid’) |
| **Classifier**  Dense (num_classes, activation = ‘softmax’) |
| **Training**  Cross-validation: Stratified 5-fold cross-validation  Optimizer: Adam (learning_rate = 0.0001) with learning rate scheduler  Loss: ['mse', 'sparse_categorical_crossentropy'], loss_weights = [0.5, 0.5]  Metrics: ['accuracy']  Epochs: 300  Batch size: 32 |

**Table S2:** CSAE spectral feature extraction and classification network parameter configuration

| **Parameters** |
| --- |
| **Filters**  Filter_1 = 32, Filter_2 = 16, Filter_3 = 8, Filter_out = 1 |
| **Encoder**  Filter_1 to Filter_3  Conv1D: (k_size = 3, padding = ' same', k_regularizer = l1(0.01)), BN, Activation = ‘elu’ |
| **Decoder**  Filter_3 to Filter_1  Conv1DTranspose: (k_size = 3, padding = ' same'), BN, Activation = ‘elu’  Conv1DTranspose: (Filter_out, k_size=3, padding = ' same'), BN, Activation = ‘elu’ |
| **Spectral Classifier**  Flatten ()  Dense: (128, activation = ‘elu’)  Dense: (num_classes, activation = ‘softmax’) |
| **Training**  Cross-validation: Stratified 5-fold cross-validation  Optimizer: Adam (learning_rate = 0.0001) with learning rate scheduler  reconstruction_loss = K.binary_crossentropy(x_true,x_pred)  sparsity_loss = K.mean(K.square(K.sum(x_pred,axis = (1,2)) − alpha))  loss = [sparse_coding_loss(alpha), ‘categorical_crossentropy’], loss_weights = [0.5,0.5]  Metrics: ['accuracy']  Epochs: 300  Batch size: 32 |
